# Acute calcific periarthritis—a commonly misdiagnosed pathology

**DOI:** 10.1007/s00256-022-04006-8

**Published:** 2022-02-11

**Authors:** Simon Dimmick, Catherine Hayter, James Linklater

**Affiliations:** Castlereagh Imaging, St Leonards NSW, 60 Pacific Highway, Sydney, Australia

**Keywords:** Acute calcific periarthritis, Radiograph, Ultrasound, MRI

## Abstract

Acute calcific periarthritis (ACP) is a self-limiting, monoarticular, peri-articular process of dystrophic mineral deposition and adjacent inflammation. Patients present with a sudden onset of pain, localised swelling, erythema, tenderness and restricted range of motion. Symptoms reduce in severity within 4–7 days and self resolve in 3–4 weeks. ACP is commonly misdiagnosed, in particular, as infective or inflammatory pathologies such as septic arthritis and gout. This condition has specific imaging findings which allows differentiation from other disorders when combined with the clinical presentation. Prompt diagnosis results in appropriate management and reduces the likelihood of unnecessary diagnostic and therapeutic procedures.

## Introduction

Acute calcific periarthritis (ACP) is a self-limiting, monoarticular, peri-articular process of dystrophic mineral deposition which was first described in 1870 by Duplay [1]. The majority of cases involve the shoulder [2, 3]. Less commonly, there may be involvement of the hip, knee, ankle, foot, elbow, wrist and fingers [1, 3–5]. As the name suggests, mineral deposition is most prevalent in a periarticular location; however, deposits within a bursa, at tendon insertions or at fascial attachments distant to the joint may also occur [6]. A history of trauma is elicited in one third of patients with acute calcific periarthritis [7]. Repetitive microtrauma in the hand (especially in manual workers) and the feet (due to footwear) is also postulated as a possible cause of ACP [8]. The true prevalence of this condition is unknown due to the self-limiting course of the condition [9].

Acute calcific periarthritis affects both males and females over a broad age range, with an average age of 45 years [7]. Some authors note that ACP involving distal articulations (elbow, wrist, hand and great toe) demonstrates a higher proportion of pre- and perimenopausal women [5, 9–11].

Due to the clinical presentation, a high proportion of cases of ACP are misdiagnosed. Rates of misdiagnosis range from 58 to 70% [9, 12, 13]. This may result in unnecessary diagnostic and management procedures, inappropriate drug therapy (particularly antibiotics) and hospital admission [9, 13]. An association between ACP and systemic diseases, such as hypothyroidism, rheumatoid arthritis, diabetes mellitus, gout and pseudogout, has been reported [14]. The objective of this article is to present the imaging findings of ACP using multiple modalities and examples from a variety of upper and lower limb joints and to discuss and differentiate ACP from other pathologies, in particular, acute septic arthritis.

## Clinical presentation, course and management

Clinically, patients present with pain, localised swelling, erythema, tenderness and restricted range of motion [10, 15, 16]. Even without treatment, the majority of patients report a reduction in symptoms within 4–7 days after the acute onset of pain and resolution in 3–4 weeks [4, 17]. Relapse of acute calcific periarthritis is uncommon [4, 13]. Few multifocal, recurrent and familial cases have been previously reported [10, 18].

Management may include local anaesthetic or corticosteroid injection, oral NSAIDs and/or use of a resting splint [8–10, 12, 19, 20]. These measures are aimed at providing both symptomatic relief and reducing the clinical course of the disease.

## Pathophysiology

The aetiology and pathophysiology of acute calcific periarthritis remain uncertain [21, 22]. Local hypoxia in critical areas of the tendon, ligament or capsule, due to poor blood flow induced by mechanical, metabolic or other factors, is postulated as the cause of calcium deposition [23].

The evolution of acute calcific periarthritis has been divided into four phases [24]. The precalcific phase (phase 1) is characterised by metaplasia of collagen fibres of the tendon into fibrocartilage. In the formative phase (phase 2), formation of calcified appetite crystals occurs which is mediated by chondrocytes. In the resorptive phase (phase 3), the accumulation of leukocytes, lymphocytes and giant cells results in formation of a calcium granuloma. The post calcific phase (phase 4) is characterised by formation of new capillaries and collagen fibres [24].

Rupture of calcific deposits and extension into an adjacent soft tissue space or bursa result in an acute crystal-induced inflammatory response, which heralds the onset of clinical symptoms [9]. Macrophages mediated by the inflammatory response then eliminate the deposits within the involved tissue via phagocytosis. On resolution, the capsule, ligament or tendon has returned to a normal structure [9, 24].

Pathological examination demonstrates deposition of calcific material into psammoma-like bodies, which is surrounded by extensive inflammatory cells, in particular, neutrophils [25, 26]. The composition of the calcific deposits on electron microscopy is controversial [15]. Previously, studies have reported the deposits to consist of calcium hydroxyapatite; hence, the term hydroxyapatite deposition disease has been used to describe the underlying disease process of calcific periarthritis and calcific tendonitis [13, 27–30]. More recent studies by Hamada and colleagues, however, have demonstrated calcium appetite [21, 22].

Chung et al. (2004) describe four macroscopic phases of calcium deposition and corresponding symptomatology. In the first phase, the calcium is contained within the tendon. Patients in phase one are asymptomatic or minimally symptomatic [31]. In the second (or mechanical) phase, there is an increase in the size of the deposit, which results in elevation of the floor of the subacromial bursa. Mass effect on the bursa may result in pain and bursitis. Deposits may extrude into the bursa (sub-bursal rupture) or complete intrabursal rupture. In phase three, there is an adhesive periarthritis and/or an adhesive bursitis. In the fourth phase (intraosseous loculation), deposits may erode into the adjacent bone at the insertion of the involved tendon or joint capsule, due to osteoclastic activity, which is postulated to be secondary to a combination of mechanical pressure and local active hyperemia [31]. Bone erosions commonly contain histiocytic infiltrate [15]. In a study by the Armed Forces Institute of Pathology, cortical erosion and extension into the intramedullary bone were demonstrated in 78% and 36% of patients respectively [32]. It is postulated that mineral particles may migrate into the marrow space even in the absence of a cortical breech, via transcortical vascular pores [15].

## Imaging findings

**Fig. 1:**
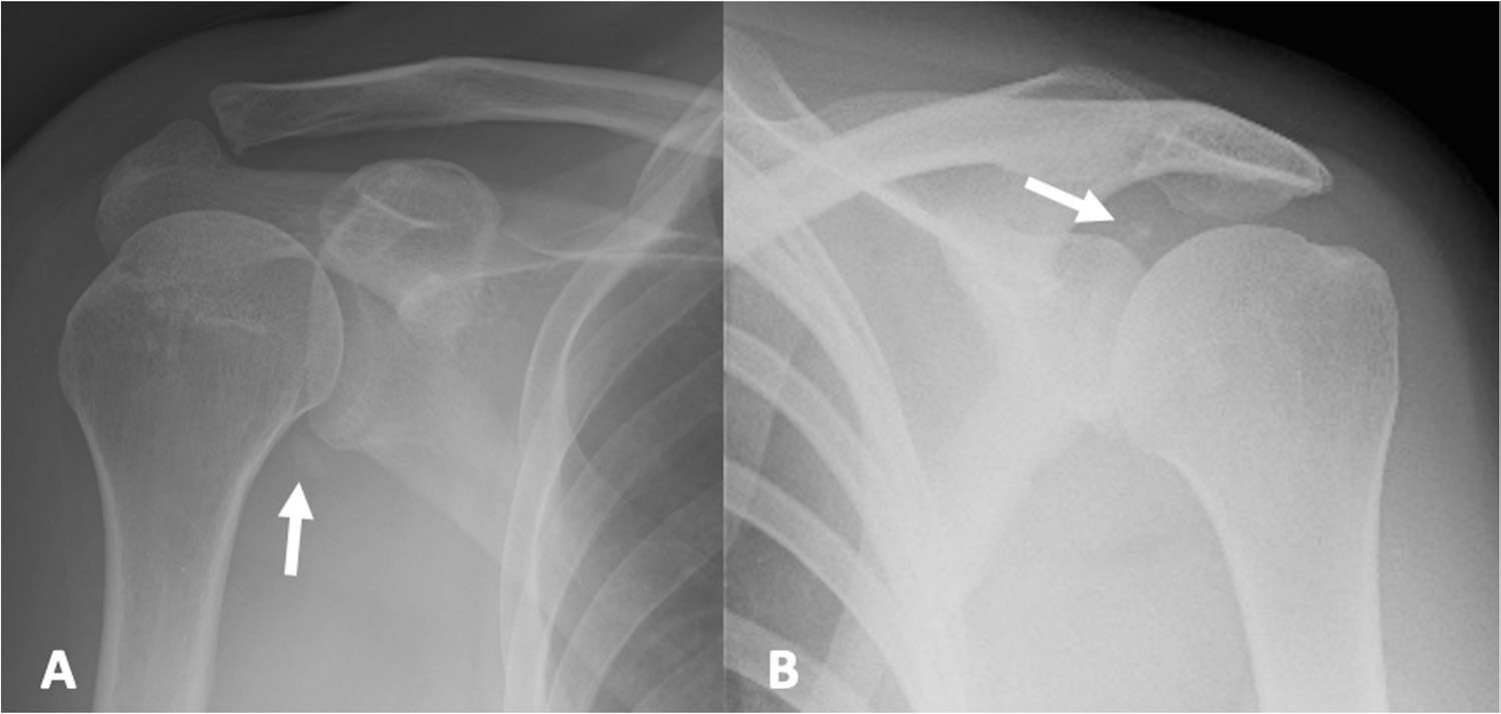
A 56-year-old female presented with a 10-day history of right shoulder pain radiating to the elbow. **A** AP radiograph of the right shoulder shows a focus of calcification in the region of the axillary recess/long head of triceps (arrow). **B** 41-year old female presented with a 3 day history of severe shoulder pain, with onset after a “pulling” injury to the shoulder. AP radiograph of the left shoulder demonstrates a rounded focus of calcification adjacent to the posterosuperior glenoid (arrow). MRI performed on the same day confirmed the diagnosis of ACP

**Fig. 2:**
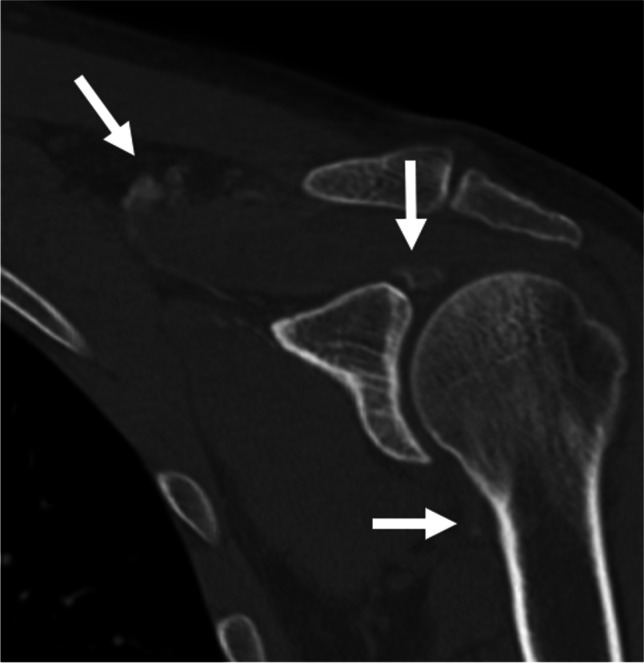
A 51-year-old male presented with a 1-week history of severe left shoulder pain after lifting weights at the gym. CT of the left shoulder with coronal reformations demonstrates a gross case of ACP, with capsular/pericapsular calcific deposits and extensive further calcification which tracks medially into the supraclavicular fat (arrows)

On radiographs, deposits of ACP are distinct, homogeneous, peri-articular densities, with no evidence of ossification (i.e. no internal trabeculae or definable cortex) that are localised to the site of symptomatology [12, 18, 33] (Fig. 1). Periarticular calcification may be located within the joint capsule or within adjacent tendons/peritendinous tissues and ligaments [34–38] (Fig. 2). There is significant variability in the size of the calcification which does not correlate with the severity of symptoms [33].

**Fig. 3:**
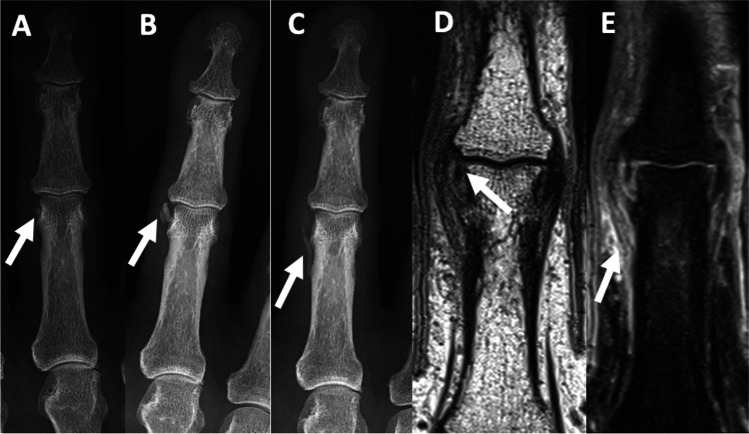
A 77-year-old male. **A**, **B** AP radiographs of the finger performed for unrelated wrist pain 3 years apart. The 3rd PIP joint was asymptomatic at this time, however, demonstrating calcification at the radial aspect of the 3rd PIP joint (arrow). The calcification in the later study is larger (arrow). **C** AP radiograph performed for acute pain involving the radial aspect of the 3rd PIP demonstrates partial resorption of the previously identified calcification and new calcification involving the radial capsule (arrow). **D** Coronal proton density MRI image demonstrates a small focus of intra-articular calcification at the radial aspect of the PIP joint; however, the capsular calcification is difficult to visualise on MRI. **E** Coronal proton density with fat-saturated image shows ligamentous/periligamentous edema involving the radial collateral ligament and capsular/pericapsular edema involving the radial joint capsule (arrow)

**Fig. 4:**
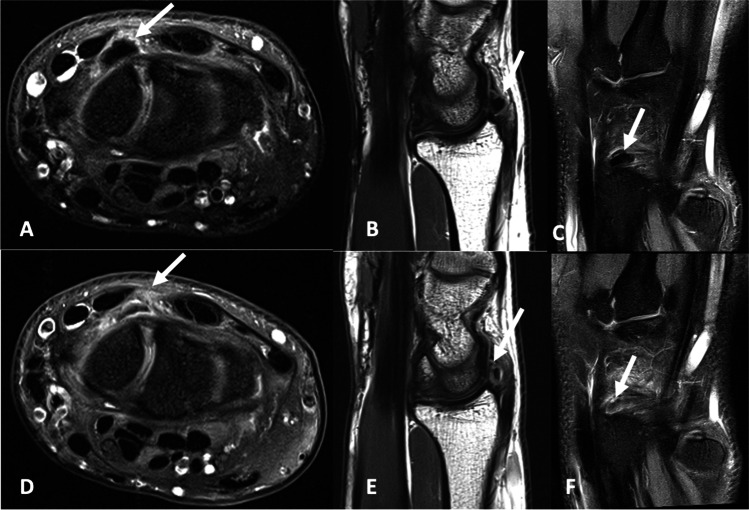
A 24-year-old male presented with dorsal wrist pain for 2 years after a fall onto an outstretched hand. **A** Axial proton density with fat saturation, **B** sagittal proton density and **C** coronal proton density with fat saturation images demonstrate ovoid calcification overlying the dorsal aspect of the proximal pole of the scaphoid involving the dorsal capsule of the radiocarpal joint and dorsal radio-triquetral ligament. **D**–**F** Corresponding images on a progress study performed 6 weeks later which shows partial resorption of the calcification

Over time, the mineralisation changes in morphology and configuration becomes less well defined and may fragment [33] (Fig. 3). Deposits usually resolve or markedly decrease within 2–3 weeks [9, 10] (Fig. 4). In some instances, calcifications may remain visible for months. Although not common, bone marrow edema may be evident in the presence of peri-articular calcification [31]. Periarticular calcific deposits may be missed or misinterpreted as accessory ossicles or avulsion fractures, particularly in the fingers and feet [15]. Acute calcific tendonitis of longus colli is a rare form of acute calcific periarthritis demonstrating prevertebral soft tissue swelling and calcification at the level of the atlanto-axial joint.

**Fig. 5:**
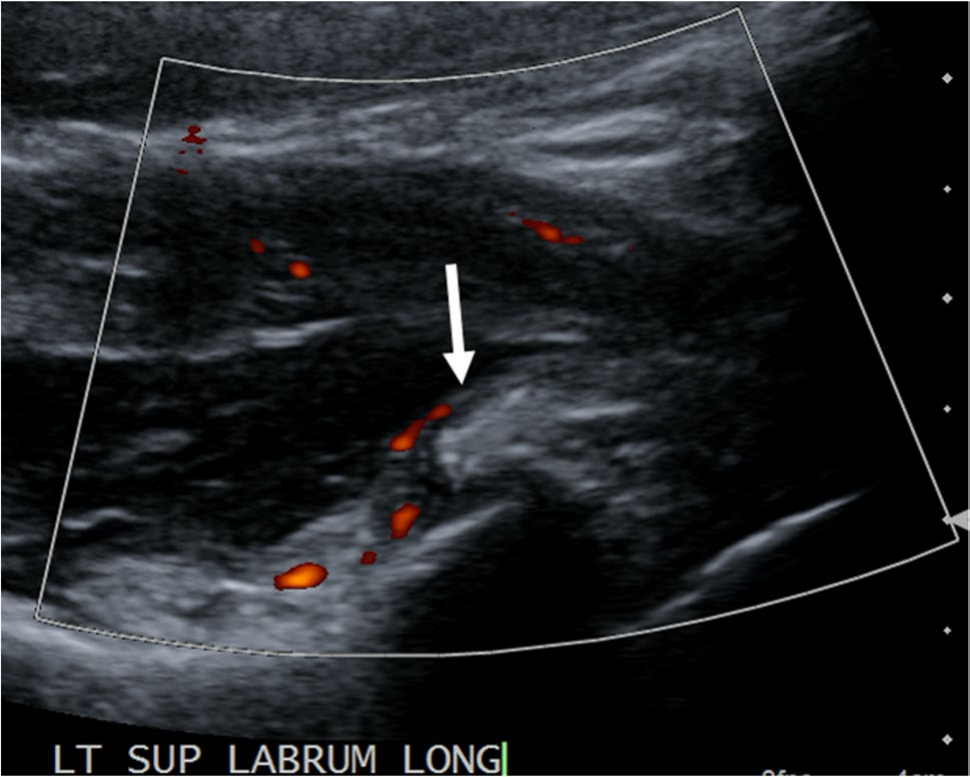
A 40-year-old male presented with a diagnosis of ACP of the left shoulder based on the findings of an MRI for ultrasound-guided corticosteroid injection. A longitudinally orientated image using color Doppler was obtained prior to the injection which demonstrates a focus of capsular calcification adjacent to the anterosuperior glenoid rim and associated capsular/pericapsular hyperemia

On ultrasound, the periarticular calcific deposits may be visible. Color Doppler may demonstrate adjacent capsular and pericapsular hyperemia (Fig. 5).

On magnetic resonance imaging, calcifications are invariably low signal intensity on all pulse sequences. Two authors [22, 28] have reported cases of calcified deposits which were hyperintense on T2-weighted sequences. Vinson postulated that this appearance in ACP is due to the liquid nature of the deposit [39]. In acute presentations, soft tissue edema is present adjacent to the calcific deposits, which, in most circumstances, correlates with the patient’s symptoms. Peri-articular calcific deposits without adjacent soft tissue edema may also occur. Review and correlation with radiographs are essential. Calcification, particularly when small, is more conspicuous on radiographs. Small calcific deposits may be easily missed on MRI without a corresponding radiograph.

**Fig. 6:**
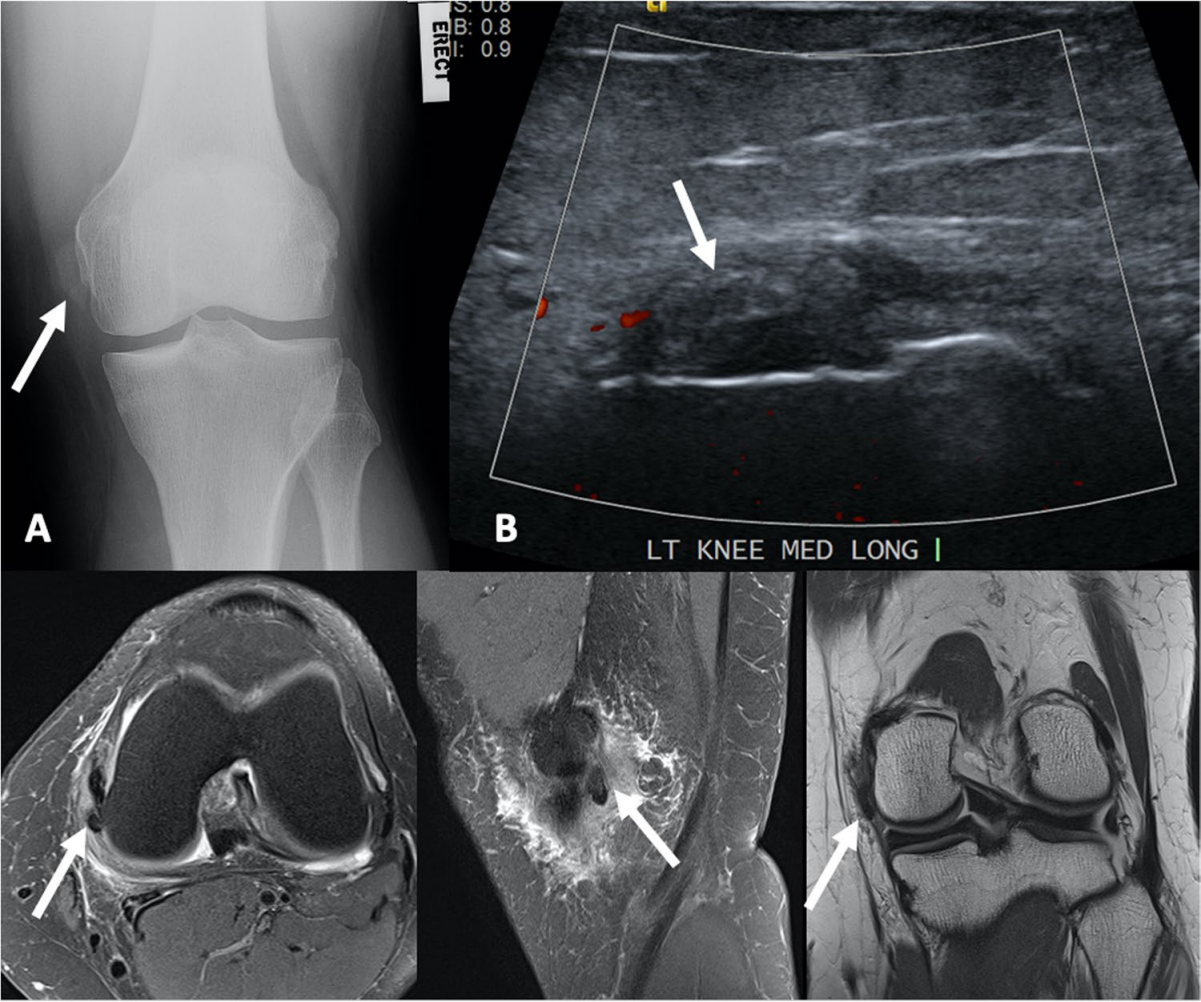
A 51-year-old female presented after a sudden onset of medial knee pain, 1 day after weight training. **A** AP weight-bearing radiograph of the left knee demonstrates an ovoid focus of calcification (arrow) adjacent to the medial condyle of the femur. **B** Longitudinally orientated ultrasound image of the medial aspect of the knee with color Doppler also shows the calcification in continuity with the posteromedial tibiofemoral joint capsule (arrow) and mild adjacent hyperemia. An MRI was performed 3 days after the plain film and ultrasound studies. **C** Axial proton density with fat saturation, **D** sagittal proton density with fat saturation and **E** coronal proton density show the capsular calcification (arrow) posterior to the medial collateral ligament, with moderate adjacent soft tissue edema

**Fig. 7:**
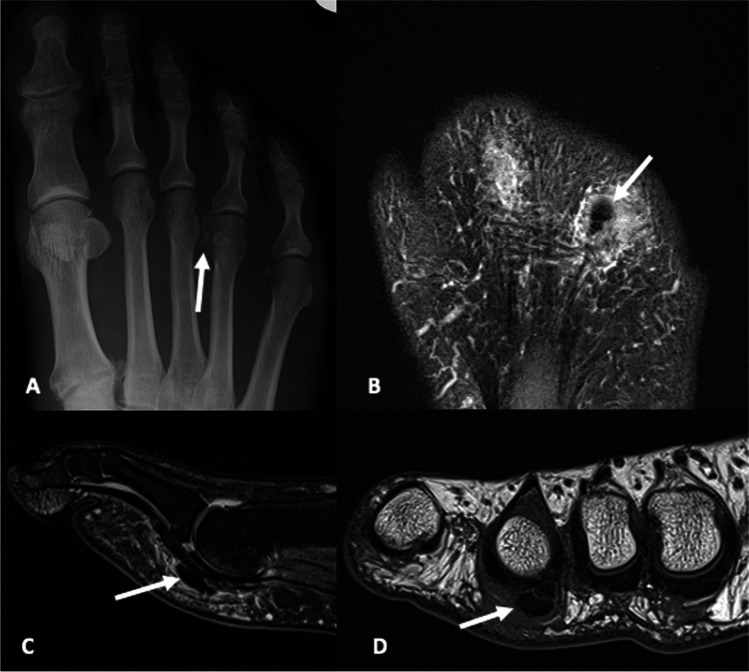
A 48-year-old male presented with pain at the plantar aspect of the right 4th metatarsophalangeal joint. **A** AP radiograph of the foot demonstrates two foci of calcification adjacent to the medial aspect of the 4th MTP joint (arrow). **B** Long axis proton density with fat saturation, **C** sagittal proton density with fat saturation and **D** short axis T1 MRI images demonstrate two foci of calcification involving the plantar/medial capsule of the 4th MTP joint (arrow) with adjacent soft tissue edema

**Fig. 8:**
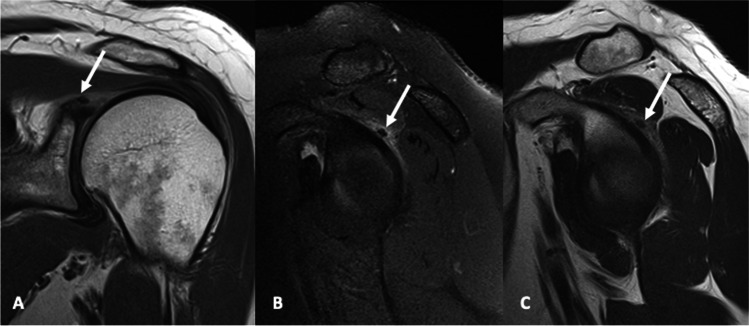
A 39-year-old male presented with a sudden onset of left shoulder pain. **A** Coronal proton density, **B** sagittal T2 with fat saturation and **C** sagittal T2-weighted images demonstrating capsular calcification involving the posterosuperior glenohumeral joint (arrow) with adjacent pericapsular edema

**Fig. 9:**
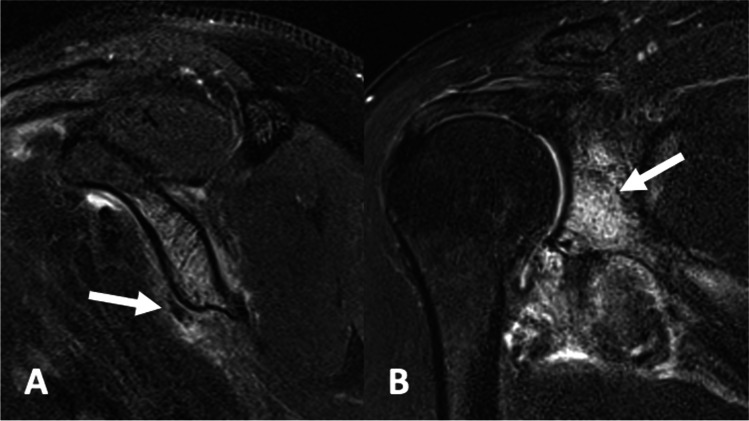
A 56-year-old female (from Fig. 1A). **A** Sagittal T2-weighted sequence with fat saturation shows diffuse bone marrow edema within the glenoid and a focus of capsular calcification adjacent to the antero-inferior glenoid (arrow). **B** Coronal proton density with fat saturation image demonstrates diffuse bone marrow edema within the glenoid (arrow) and florid capsular/pericapsular edema

**Fig. 10:**
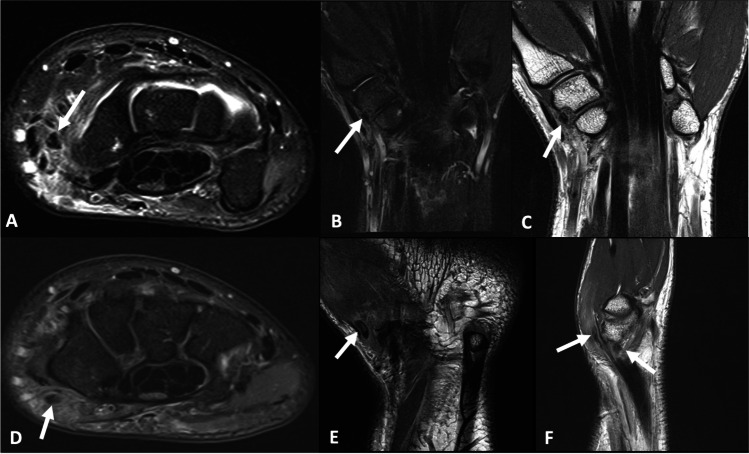
A 34-year-old female presented with a sudden onset of radial sided right wrist pain. **A** Axial proton density with fat saturation, **B** coronal proton density with fat saturation and **C** coronal proton density images demonstrate a focus of calcification involving the radial aspect of the scapho-trapezium joint (arrow), with associated pericapsular edema and mild bone marrow edema within the radial aspect of the trapezium and distal pole of the scaphoid. **D** Axial proton density with fat saturation and **E** coronal proton density images demonstrate a further focus of pericapsular calcification (arrow), volar to the capsular calcification of the scapho-trapezium joint. **F** Sagittal proton density image shows both foci of calcification (arrows)

**Fig. 11:**
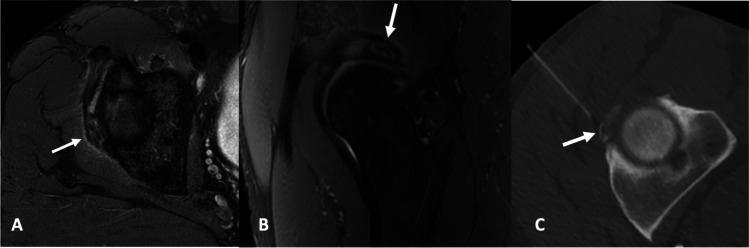
A 47-year-old male presented with an acute onset of severe anterior hip pain. **A**, **B** Axial and sagittal proton density sequences with fat saturation demonstrate superolateral intra-labral calcification (arrow) and adjacent mild paralabral edema. **C** Axial CT image obtained during an image-guided corticosteroid injection best demonstrates the intra-labral calcification (arrow)

The edema may be capsular or pericapsular, bursal, ligamentous/peri-ligamentous or peritendinous [31] (Figs. 6, 7 and 8). Associated bone marrow edema, cortical erosion and intra-osseous extension are uncommon (Fig. 9). Bone scan may demonstrate uptake corresponding to the site of calcific deposits [8, 12]. Occasionally, capsular and pericapsular calcific deposits may be multi-focal (Fig. 10). CT or ultrasound may be used to perform imaging-guided injections of corticosteroid and anaesthesia (Fig. 11).

## Differential diagnoses

Acute calcific periarthritis has a high rate of misdiagnosis as it may clinically mimic other pathology [13]. The most commonly misdiagnoses include infective and inflammatory processes, arthropathies or less likely neoplasia [4, 15].

Acute infection may be excluded based on the imaging findings. In most circumstances, calcification such as occurring in ACP is not present in acute infection, unless there is pre-existing calcification secondary to superimposed chronic renal disease or crystalline arthropathy. Soft tissue calcification is a late manifestation of infection which may be visualised weeks to months after onset [40]. Although superimposed infection has been reported in the setting of acute calcific periarthritis, this is rare [4].

The monoarticular nature of acute calcific periarthritis and that it does not involve the joint proper may assist in differentiating this entity from other inflammatory and erosive arthropathies. Crystal arthropathies such as gout and calcium pyrophosphate dihydrate (CPPD) crystal deposition disease may also be differentiated radiologically from acute calcific periarthritis. Similar to infection, calcification is present in the intermediate and late stages of chronic gout as a tophus. Tophi however are associated with a soft tissue mass. Also pathognomonic for gout are juxta-cortical “punched out” erosions with sclerotic margins. These erosions may also have an overhanging edge of cortex [41]. Gout tends to have and asymmetric polyarticular distribution [41]. In many circumstances, patients who present with an acute attack of gout will have a pre-existing diagnosis of gout and will report a history of recurrent exacerbations.

CPPD is estimated to afflict 5% of the population and may present with acute exacerbations and similar appearing calcifications within the soft tissues [41]. Chondrocalcinosis, which is the deposition of CPPD crystals into fibrous or hyaline cartilage is not exclusive to CPPD, however, is not present in ACP [341]. Calcifications associated with CPPD arthropathy are reported to be more linear or elongated than those seen in ACP and hydroxyapatite deposition disease [18]. Crystal deposition may also be present in ligaments, tendons and bursae.

Other radiographic findings which may be seen in CPPD include a bilateral distribution, uniform joint space loss, subchondral new bone formation and intra-osseous cysts which may be more prominent than those associated with osteoarthritis [41].

Many of the systemic arthritides (such as systemic lupus erythematosus (SLE) and psoriatic arthritis) may also be associated with calcifications [41]. In each of these pathologies, the calcifications tend to be multiple. Additional findings in SLE include juxta-articular osteoporosis, subluxations/dislocations and osteonecrosis. In psoriatic arthritis, there is a bilateral asymmetrical distribution, with joint space loss, bone proliferation and erosions (“pencil in cup”). The most commonly involved joints in order of frequency are the hands, feet, sacroiliac joints and spine, whereas the most commonly involved joint in ACP is the shoulder [41].

Metastatic calcification and collagen vascular disease (such as scleroderma) may mimic the calcifications of ACP; however, the clinical presentation in these conditions differs to that of ACP [31]. Metastatic periarticular calcifications may be due to end-stage renal disease, hypoparathyroidism, tumoral calcinosis, vitamin D intoxication and sarcoidosis [39]. A cortex and internal trabeculation, characteristics of heterotopic ossification, are not present in ACP [23, 39]. Metabolic disease such as hypophosphatasia has been reported as an uncommon cause of ACP.

## Limitations

Histopathological correlation for the cases presented in this article has not been obtained. Each case was diagnosed with acute calcific periarthritis after consensus with the reporting musculoskeletal radiologist and the referring physician and subsequent clinical follow-up.

## Conclusion

Acute calcific periarthritis is an uncommon but important diagnosis. Greater awareness of the clinical presentation and radiological findings will result in prompt diagnosis and management and more rapid resolution. This will also reduce further unnecessary investigations, potential biopsy and inappropriate management.
